# An RNA sequencing dataset from a porcine immortalized pre-adipocyte cell line

**DOI:** 10.1016/j.dib.2025.112074

**Published:** 2025-09-18

**Authors:** Susanna E. Riley, Thomas Thrower, Seungmee Lee, Cristina L. Esteves, F. Xavier Donadeu

**Affiliations:** Division of Translational Bioscience, The Roslin Institute and Royal (Dick) School of Veterinary Studies, University of Edinburgh, Edinburgh, UK

**Keywords:** Adipogenesis, Cell Immortalization, Mesenchymal stem/stromal cells, Pre-adipocyte, Pig

## Abstract

There is a significant need for livestock cell lines that can be robustly expanded in culture while maintaining their functional characteristics, both for use as *in vitro* models to understand animal physiology and for industrial applications such as in the emerging sector of cellular agriculture. Here we describe RNA sequencing datasets from a spontaneously immortalized pre-adipocyte line that was derived through serial passaging of porcine adipose-derived mesenchymal stromal cells (MSCs). This cell line, known as FaTTy, is unique in that it displays enhanced adipogenic capacity during long-term culture, characterised by a close to 100% differentiation efficiency and the ability to generate mature adipocytes. Bulk RNA sequencing was performed from FaTTy and parental MSCs. We present analysis of the raw files and bioinformatics analyses, including differential gene expression. These data provide valuable insight on the mechanisms of cell immortalization and the unique phenotype of the FaTTy cell line, with distinctive advantages as a prospective cell source for cultivated fat manufacture.

Specifications Table

**Table:** 

Subject	Biology
Specific subject area	Mesenchymal stem cell biology and immortalization, adipogenic determination
Type of data	Table, Graph, Figure Raw and analysed bulk RNA-seq data
Data collection	Total RNA was extracted from cells in exponential growth phase using TRIzol and QIAGEN RNAeasy mini kit. RNA sequencing was performed with the Illumina NovaSeq 6000 platform. Software used for bioinformatic analyses were bcl2fastq, FastQC, Trimmomatic, STAR aligner, Subread, DESeq2
Data source location	The Roslin Institute and Royal (Dick) School of Veterinary Studies, University of Edinburgh, Edinburgh, UK
Data accessibility	Repository name: NCBI’s Gene Expression Omnibus (GEO) Data identification number: Series GSE266992, BioProject PRJNA1109311 Direct URL to data: https://www.ncbi.nlm.nih.gov/geo/query/acc.cgi?acc=GSE266992
Related research article	T. Thrower, S.E. Riley, S. Lee, C.L. Esteves, F.X. Donadeu. A unique spontaneously immortalized cell line from pig with enhanced adipogenic capacity - a foundational tool for cellular agriculture. npj Science of Food (2025) 9:52. https://doi.org/10.1038/s41538-025-00413-y

## 1 Value of the Data

• This dataset provides valuable insight into the molecular (transcriptomic) changes underlying the highly unique phenotype of the FaTTy cell line, contributing to an understanding of the molecular mechanisms of 1) spontaneous cell immortalization and 2) acquisition and long-term maintenance of adipogenic capacity by MSCs
• Mining this dataset will provide abundant information for hypothesis testing on the above mechanisms, and as such the data has been made available to the wider research community
• The information contained herein will be particularly useful to those interested in using the cell line as a research tool into livestock adipose biology or for cellular agriculture applications.
• The data is organized such that both the RNA sequencing raw data files and processed files are available for use in multiple workflows and bioinformatic analyses other than those already published

## 2 Background

Because of its unique proliferative and adipogenic capacities, and non-genetically modified (GM) nature, the FaTTy line holds unprecedented promise as a cell source for industrial cultivated fat production as well as *in vitro* model to understand adipose tissue development in pig. The emergence of FaTTy could be attributed to an exceptionally rare event in culture, with FaTTy being doubly unique in that it resulted from spontaneous immortalization of a progenitor MSC population, and displays increased adipogenic capacity over time. In order to gain insight into the molecular basis of this unique phenotype, we performed bulk RNA sequencing before and after cell immortalization (parental MSCs and FaTTy, respectively), as part of wider phenotypic and genomic characterisation of the cell line, as described in [1]. The differentially expressed gene profiles thus obtained revealed candidate gene and pathways potentially involved in the development of FaTTy’s unique characteristics, in terms of both cell proliferation and differentiation capacity.

## 3 Data Description

RNA from wild-type (WT) parental MSCs at population doubling (PD) 15 (n=4, labelled MSC_A to MSC_D in GEO [2]) and immortalized FaTTy cells at PD72 (n=4, labelled FaTTy_A to FaTTy_D in GEO [2]) were sequenced. Raw fastq files for each sample and the gene counts with associated Ensembl ID and HGNC symbols are deposited in GEO [2].

**Fig. 1:**
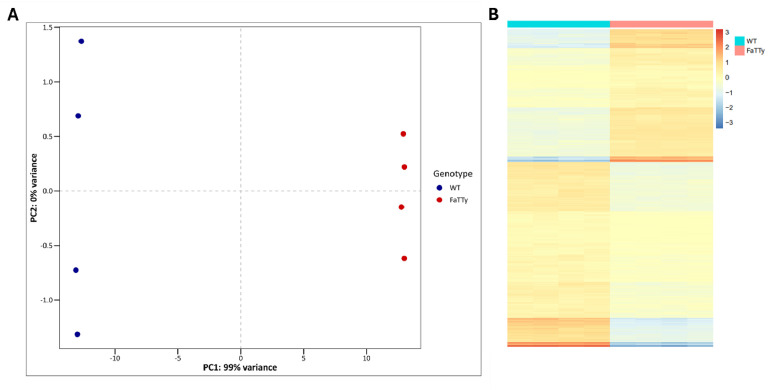
RNA sequencing dataset overview. (A) PCA plot showing parental MSC (blue) and FaTTy (red) samples clustered according to the degree of similarity in their gene expression profiles. PC1 denotes 99.37 % of all variance, whilst PC2 denotes 0.25 % of all variance. Each point represents one sample. (B) Heatmap showing relative changes in gene expression in FaTTy and parental cells. DEGs with *p-adj* <0.05 and -1 ≥ log2 fold change ≥ 1 were used, whereby the transformed counts normalised to the mean for each gene were plotted in a heat map to show changes in expression, with a negative value (blue) representing a decrease in gene expression and a positive value (red) representing an increase in gene expression.

The number of reads and mean quality score for each sample, along with the GEO accession number, are displayed in Table 1. Fig. 1A shows results of principal component analysis (PCA), demonstrating clear separation between WT and FaTTy cells. Differential gene expression analysis between FaTTy and parental cells revealed a total of 1280 differentially expressed genes (DEGs, *p-adj* < 0.05 and -1 ≥ log2 fold change ≥ 1). Of these, 572 genes were upregulated and 708 were downregulated in FaTTy cells (see Supplementary Data 1 in [1] for a full list of genes). Fig. 1B shows DEGs as a heatmap, again showing clear separation between the two cell types.

**Table 1:** Read number, quality score, and GEO accession number of samples included in this dataset.

Sample	Read number	Mean Quality Score	Name in GEO repository	GEO accession number
WT_A	28,453,818	36.00	MSC_A	GSM8257847
WT_B	40,829,997	35.93	MSC_B	GSM8257848
WT_C	30,051,030	35.92	MSC_C	GSM8257849
WT_D	29,447,622	35.93	MSC_D	GSM8257850
FaTTy_A	22,469,254	36.01	FaTTy_A	GSM8257851
FaTTy_B	23,028,696	35.98	FaTTy_B	GSM8257852
FaTTy_C	30,214,122	35.91	FaTTy_C	GSM8257853
FaTTy_D	20,761,021	35.85	FaTTy_D	GSM8257854

Samples with a quality score of >30 (*i.e.*, <0.1 % error rate) were considered to be of high quality [3]. A-D denotes sample replicates.

## 4 Experimental Design, Materials and Methods

### 4.1 RNA extraction

RNA was extracted from cells before and after immortalization, *i.e.*, parental MSCs (PD15) and FaTTy cells (PD72), grown as detailed in [1]. All cells were collected at exponential growth phase by detachment with Trypsin-EDTA (25,200–056, Gibco), washed in PBS and resuspended in TRIzol reagent (15,596,026, Invitrogen) before storage at -80°C.

Following thawing, 1-bromo-3-chloropropane (B9673, Sigma-Aldrich) was added, mixed for at least 15 s, and incubated for 3 min at room temperature. Samples were then centrifuged for 15 min at 12,000x*g* at 4°C. The upper aqueous layer was removed and processed for RNA extraction using a RNeasy Mini kit (74,104, Qiagen) as per the manufacturer’s instructions. RNA was eluted in nuclease-free water (1039,498, Qiagen) and RNA quality and concentration were determined via Nanodrop (ND-1000). Samples were stored at -80°C.

### 4.2 RNA sequencing and data processing

RNA sequencing and initial data processing up to generation of gene counts was performed by GENEWIZ UK, Azenta Life Sciences (Takeley, Essex, UK). The RNA sequencing library was prepared using the NEBNext Ultra II Directional RNA Library Prep Kit (New England Biolabs) as per manufacturer’s instructions. Briefly, this involved, sequentially, mRNA enrichment, RNA fragmentation and random priming steps, followed by cDNA synthesis and dUTP incorporation. This was in turn followed by adenosine tailing and, finally, adaptor ligation, dUTP strand degradation and PCR amplification. The library was validated using DNA Kit on an Agilent 5600 Fragment Analyzer (Agilent Technologies), and quantified on a Qubit 4.0 Fluorometer (Invitrogen), before sequencing on the Illumina NovaSeq 6000 platform, as per manufacturer’s instructions, with a 2 × 150 bp Paired End configuration (v15). Image analysis and base calling was performed using NovaSeq Control Software (v.1.7).

Fastq files were generated from raw sequencing data using Illumina bcl2fastq (v2.20) software, allowing one mismatch for index sequence identification. Following quality control using FastQC, sequence reads were trimmed to remove possible adapter sequences and nucleotides with poor quality, using Trimmomatic (v0.36). The trimmed reads were mapped to the Sus scrofa 10.2 reference genome available on ENSEMBL using the STAR aligner v.2.5.2b The STAR aligner is a splice aligner that detects splice junctions and incorporates them to help align the entire read sequences. BAM files were generated as a result of this step.

Unique gene hit counts were calculated by using featureCounts from the Subread package v.1.5.2. The hit counts were summarized and reported using the gene_id feature in the annotation file. Only unique reads that fell within exon regions were counted.

### 4.3 Differential expression analyses

Differential expression calculations were performed using the DESeq2 package (v1.42.1) in R (v4.3.2/v4.3.3), removing genes with 0 reads and using WT samples as a reference. For the PCA plot (Fig 1A), the DESeq object was transformed using the vst() function. For the heatmap (Fig 1B), the DESeq object was transformed using the rlog() function, and only DEGs were plotted. DEGs were defined as those with absolute log2 fold change values of ≥ 1, and p-value cut-off of *p-adj* < 0.05. Analysis was performed using R packages rlog (v0.1.0), pheatmap (v1.0.12), RColorBrewer (v1.1–3), dplyr (v1.1.4), tidyverse (v2.0.0), ggplot2 (v.3.5.0), and ggthemes (v5.1.0).For both heatmap and PCA generation, code is made available in Supplementary Information file.

## Limitations

The authors note that reference genome Sus scrofa 10.2 (Ensembl) does not include annotation of sex chromosomes. Therefore, this dataset is limited because it does not reflect loss of Y chromosome as noted experimentally [1]. Re-analysis using Sus scrofa 11.1 (Ensembl) is recommended to investigate this.

## Ethics Statement

The authors have read and follow the ethical requirements for publication in Data in Brief and confirm that the current work does not involve human subjects, animal experiments, or any data collected from social media platforms.

## CRediT authorship contribution statement

**Susanna E. Riley:** Formal analysis, Visualization, Data curation, Writing – original draft, Writing – review & editing. **Thomas Thrower:** Investigation, Writing – review & editing. **Seungmee Lee:** Investigation. **Cristina L. Esteves:** Conceptualization, Writing – review & editing. **F. Xavier Donadeu:** Writing – review & editing, Conceptualization, Supervision, Project administration, Funding acquisition.

## Data Availability

GEO)RNA sequencing dataset of spontaneously immortalised pre-adipocyte line from pig (Original data)

## Appendix Supplementary materials

Image, application 1
