# Supplementary material for: An RNA sequencing dataset from a porcine immortalized pre-adipocyte cell line
Source: Data Brief. 2025 Sep 18;63:112074. doi: 10.1016/j.dib.2025.112074 (PMC12514535; doi:10.1016/j.dib.2025.112074)
Supplement: Supplementary file 1 [file mmc1.docx]

**Supplementary information**

***Scripts for generating PCA/Heatmap***

*Note: File names and paths have been reworded to enable generic use.*

##PCA

#Load programmes################################################################

library(DESeq2)

library(tidyverse)

library(dplyr)

library(RColorBrewer)

library(rlog)

library(ggplot2)

library(ggthemes)

rm(list=ls())

#Read in files ########################################################

htseq <- read.csv(file = "raw_counts_names_FaTTy.csv",

header=TRUE, row.names = 1)

colData <- read.csv(file = " Sample_info.csv",

header=TRUE, row.names=1)

#Wrangle data###################################################################

##Change row numbers to name

htseq <- htseq[-c(9)]

head(htseq)

colData

##Load for aesthetics

RNAseq <- c("darkblue", "red3")

#DESeq##########################################################################

##Check row names in colData match column names in htseq

all(colnames(htseq) %in% rownames(colData))

##Check in same order

all(colnames(htseq) == rownames(colData))

##Construct a DESeq2 dataset object

dds <- DESeqDataSetFromMatrix(countData = htseq,

colData = colData,

design = ~ Genotype)

dds

##Remove rows with 0 reads total

keep <- rowSums(counts(dds)) > 0

dds <- dds[keep,]

dds

##Tell DESeq to use Wt as reference

dds$Genotype <- relevel(dds$Genotype, ref = "WT")

##Run DESeq

dds <- DESeq(dds)

res <- results(dds)

##Transform data for plotting

vsd <- vst(dds, blind=FALSE)

#PCA plot#######################################################################

##Wrangle data

pcaData <- plotPCA(vsd, intgroup=c("Genotype"), returnData=TRUE)

percentVar <- round(100 * attr(pcaData, "percentVar"))

##Plot

PCAplot <- ggplot(pcaData, aes(PC1, PC2, color=Genotype)) +

geom_point(size=3) +

theme_base() +

xlab(paste0("PC1: ",percentVar[1],"% variance")) +

ylab(paste0("PC2: ",percentVar[2],"% variance")) +

theme(text = element_text(size = 14),

axis.text.x = element_text(colour="black"),

axis.text.y = element_text(colour="black"),

axis.title.x = element_text(face="bold"),

axis.title.y = element_text(face="bold")) +

geom_hline(yintercept=0, lty=2, colour = "Grey") +

geom_vline(xintercept=0, lty=2, colour = "Grey") +

scale_colour_manual(values=RNAseq)

PCAplot

#Save plot##############################################################

ggsave(filename="PCAplot_FaTTy.png",

plot=PCAplot, width = 9, height = 7, dpi = 1200)

##Heatmap

#Load programmes#################################################################

library(DESeq2)

library(tidyverse)

library(pheatmap)

library(dplyr)

library(RColorBrewer)

library(rlog)

rm(list=ls())

#Read in files########################################################

htseq <- read.csv(file = "raw_counts_names_FaTTy.csv",

header=TRUE)

colData <- read.csv(file = "Sample_info.csv",

header=TRUE, row.names=1)

#Read in files from work########################################################

htseq <- read.csv(file = "raw_counts_names_FaTTy.csv",

header=TRUE)

colData <- read.csv(file = "Sample_info.csv",

header=TRUE, row.names=1)

#Wrangle data###################################################################

##Change row numbers to name

htseq <- htseq %>%

distinct(Name, .keep_all = TRUE) %>%

column_to_rownames(var = "Name")

htseq <- htseq[-c(1)]

head(htseq)

colData

#DESeq##########################################################################

##Check row names in colData match column names in htseq

all(colnames(htseq) %in% rownames(colData))

##Check in same order

all(colnames(htseq) == rownames(colData))

#DESeq2 analysis

##Construct a DESeq2 dataset object

dds <- DESeqDataSetFromMatrix(countData = htseq,

colData = colData,

design = ~ Genotype)

dds

##Remove rows with 0 reads total

keep <- rowSums(counts(dds)) > 0

dds <- dds[keep,]

dds

##Tell DESeq to use Wt as reference

dds$Genotype <- relevel(dds$Genotype, ref = "WT")

##Run DESeq

dds <- DESeq(dds)

res <- results(dds)

##Transform data for downstream plotting

rld <- rlog(dds, blind=FALSE)

##Make df for labelling

df <- as.data.frame(colData(dds)[,"Genotype"])

colnames(df) <- "Cell type"

##Order the results according to absolute value of test statistic (correlates perfectly with p-values)

res_ordered <- res[order(abs(res$stat), decreasing=TRUE),]

##Construct a results matrix

deseq <- `rownames<-`(cbind("LogFold"=res_ordered$log2FoldChange,

"P.Value"=res_ordered$pvalue,"P.Adj"=res_ordered$padj),

rownames(res_ordered))

#Plot overall heatmap###########################################################

##Subset significant genes, according to logFC threshold and adjusted p values

top_DE_genes <- rownames(deseq)[which(deseq[,"P.Adj"] < 0.05 & abs(deseq[,"LogFold"]) > 1)]

##Find values for top DE genes in rld dataset for plotting

mat <- assay(rld)[top_DE_genes, ]

mat <- mat -rowMeans(mat)

##Ensure mat colnames = df rownames

colnames(mat) <- str_sub(colnames(mat))

rownames(df) <- colnames(mat)

##Heatmap

overall <- pheatmap(mat, cluster_rows=TRUE, show_rownames=FALSE,

show_colnames=FALSE, cluster_cols=FALSE,

annotation_col=df, annotation_legend = TRUE, annotation_names_col = FALSE,

treeheight_row = 0)

#Top 30 DE genes heatmap########################################################

##Make df of top genes

deseq_df <- as.data.frame(deseq) %>%

filter(P.Adj < 0.05) %>%

filter(LogFold > 1 | LogFold < (-1)) %>%

rownames_to_column("ID")

##Order df by lfc

deseq_df <- deseq_df[order(deseq_df$LogFold, decreasing=TRUE),]

head(deseq_df)

tail(deseq_df)

#Take top and bottom 15 genes

deseq_df_head <- deseq_df %>%

slice_head(n=15)

deseq_df_tail <- deseq_df %>%

slice_tail(n=15)

deseq_df_top <- rbind(deseq_df_head, deseq_df_tail)

top_DE_genes_30 <- deseq_df_top$ID

##Find values for top DE genes in rld dataset for plotting

mat_top <- assay(rld)[top_DE_genes_30, ]

mat_top <- mat_top -rowMeans(mat_top)

##Create df with gene names

namesmat_top <- as.data.frame(mat_top)

head(namesmat_top)

##Ensure mat_top colnames = df rownames

colnames(namesmat_top) <- str_sub(colnames(namesmat_top))

rownames(df) <- colnames(namesmat_top)

##Heatmap

top <- pheatmap(namesmat_top, cluster_rows=TRUE, show_rownames=TRUE,

show_colnames=FALSE, cluster_cols=FALSE,

annotation_col=df, annotation_legend = TRUE, annotation_names_col = FALSE,

treeheight_row = 0)

#Save DESeq results#####################################################

deseq_save <- as.data.frame(deseq)

head(deseq_save)

write.csv(deseq_save,

file = "DEGs_all.csv",

row.names = TRUE)

#Save heatmaps at home##########################################################

dev.off()

pdf(file="heatmap.pdf")

overall

dev.off()

pdf(file="heatmap_top.pdf")

top

dev.off()
